# Immunological Dynamics Associated with Direct-Acting Antiviral Therapies in Naive and Experimented HCV Chronic-Infected Patients

**DOI:** 10.1155/2019/4738237

**Published:** 2019-11-04

**Authors:** Grenda Leite Pereira, Andréa Monteiro Tarragô, Walter Luiz Lima Neves, Pedro Vieira da Silva Neto, Priscila Sarmento de Souza, Juliana dos Santos Affonso, Keyla Santos de Sousa, Jéssica Albuquerque da Silva, Allyson Guimarães Costa, Flamir da Silva Victoria, Marilu Barbieri Victoria, Adriana Malheiro

**Affiliations:** ^1^Universidade Federal do Amazonas (UFAM), Manaus, AM, Brazil; ^2^Fundação Hospitalar de Hematologia e Hemoterapia do Amazonas (HEMOAM), Manaus, AM, Brazil; ^3^Instituto de Pesquisas Leônidas & Maria Deane, FIOCRUZ-Amazônia, Manaus, AM, Brazil; ^4^Universidade do Estado do Amazonas (UEA), Manaus, AM, Brazil; ^5^Fundação de Medicina Tropical Doutor Heitor Vieira Dourado (FMT-HVD), Manaus, AM, Brazil

## Abstract

The therapeutic strategies used in the treatment of hepatitis C are essentially based on the combination of direct-acting antiviral agents (DAAs). This therapy has been shown to be very effective in relation to patient adherence to treatment and has shown high rates of sustained virological response (SVR). However, the immunological dynamics of patients infected with HCV is poorly understood. This fact led us to investigate the immune system of naive and experienced patients, who we followed before the therapy and three months after the end of treatment. In this study, 35 naive and experienced Brazilian patients with chronic hepatitis C and 50 healthy donors (HD group) were studied. The analysis of the soluble immunological biomarkers was performed using the flow cytometry methodology. The SVR rate was >90% among the 35 patients. Before treatment, correlations in the naive HCV group demonstrated a mix of inflammatory response occurring with moderate correlations between chemokines, inflammatory cytokines, and Th2 profile, with a strong regulation between IL-10 and IL-17A. On the other hand, experienced patients demonstrated a poor interaction between cytokines, chemokines, and cells with a strong correlation between IL-10, IL-6, CXCL-10, and CD8^+^ besides the interactions between IFN-*γ* and IL-4. Furthermore, naive and experienced patients seem to have a distinct soluble biomarker profile; therefore, a long-term follow-up is needed to evaluate patients treated with DAAs.

## 1. Introduction

Hepatitis C is a chronic, inflammatory, slow-course, progressive, and asymptomatic liver disease caused by the hepatitis C virus (HCV). The global prevalence of chronic HCV infection has been estimated at 71 million people worldwide and can be regarded as a serious public health problem [1].

HCV infection causes deregulation of the host's immune response, favoring the chronicity of liver disease through altered and/or exacerbated signaling of proinflammatory cytokines and chemokines that may compromise the viral clearance and promote the development of hepatocellular carcinoma. In this context, it is noted that there is a great need to identify possible early biomarkers that help in the identification of the predisposition to the development of the most severe form of liver disease [2].

However, there are some factors that may be predictive of persistent infection and progression to more severe forms of the disease, such as advanced age, male sex, diabetes, obesity, HBV/HIV virus coinfection, hepatic steatosis, and alcoholism. These may contribute to the susceptibility and development of the chronic and inflammatory disease and may give the patient a greater predisposition to developing the disease [3].

Currently, there is no effective prophylactic or therapeutic vaccine to prevent HCV replication due to high virus variability and complexity [4]. However, a promising IFN-free treatment was approved by the Ministry of Health for the treatment of chronic hepatitis C. The new therapeutic modalities are aimed at expanding access to care for the disease in order to improve the quality and life expectancy of patients. DAAs have been recommended to treat hepatitis C by targeting nonstructural hepatitis C virus proteins such as NS3-4A, NS5A, and NS5B and act by inhibiting the replicative cycle of the virus. This new regimen has fewer side effects, is a short-term treatment, and has 90% cure rate [5].

This treatment shows a substantial improvement in sustained virological response (SVR) by DAAs that inhibit HCV replication [6]. Changes in the profiles of inflammatory mediators are observed even after viral elimination, which may contribute to these patients becoming vulnerable to the development of hepatocellular carcinoma. Thus, the present study is aimed at analyzing the circulating levels of cytokines, chemokines, and cell profiles in patients with chronic hepatitis C submitted to DAA therapy.

## 2. Materials and Methods

### 2.1. Study Design

A prospective longitudinal study was conducted with Brazilian patients diagnosed with chronic hepatitis C who received interferon-free treatment regimens. This study was approved by the Ethical Review Committee at Fundação Hospitalar de Hematologia e Hemoterapia do Amazonas (HEMOAM) (1.405.965/2015). Patients with chronic hepatitis C were selected by spontaneous demand from the Hepatopathology Clinic of the Fundação de Medicina Tropical-Dr. Heitor Vieira Dourado (FMT-HVD) in the period from April 2016 to March 2018 to receive the treatment with DAAs. The individuals in the healthy donor (HD) group were recruited at the Fundação Hospitalar de Hematologia e Hemoterapia do Amazonas (HEMOAM) from May 2016 to September 2016.

Study participants were invited to participate in the study by signing the Informed Consent Form (ICF). Some clinical and biochemical data were obtained from FMT-HVD patients' electronic medical records, including fibrosis grade, viral load, HCV genotype, and transaminases (ALT/AST).

Patients with chronic hepatitis C were monitored before starting treatment with DAAs, as well as three months after the end of treatment for SVR. Treatment consisted of daily doses of Sofosbuvir (400 mg) in combination with Simeprevir (150 mg) or Daclatasvir (60 mg) for 12 or 24 weeks. No patient included in this study received IFN during treatment with DAAs. Diagnosis, prescription, and clinical follow-up were performed by the clinical group at the Fundação de Medicina Tropical-Dr. Heitor Vieira Dourado (Manaus, Brazil) according to hepatitis C protocol approved by the Brazilian Ministry of Health [7].

This study included 85 individuals living in Manaus, Amazonas, Brazil. Subjects were categorized into two subgroups referred to as patients infected with hepatitis C virus (HCV) and uninfected individuals framed as healthy donors (HD). The group of patients diagnosed with chronic hepatitis C comprised of 35 patients, with positive serology for HCV infection (anti-HCV IgG detected by ELISA and RNA detected by NAT). Patients coinfected with hepatitis A, B, D, and E and human immunodeficiency virus (HIV) and those who had begun treatment for HCV infection were excluded from the study. The healthy donors (HD) consisted of 50 healthy individuals of both sexes, recruited as candidates for blood donations at HEMOAM. Individual HD were randomly included and submitted to a serological screening at HEMOAM, recommended to monitor blood-borne infections by Brazilian Blood Donor Bank Authorities. Serological analysis was performed for the hepatitis B and C viruses, HIV, HTLV, and Chagas' Disease.

### 2.2. Analysis of HCV Viral Load

The HCV genomic RNA assay (RNA-HCV) was performed on Amplicor RT-PCR (Roche, NJ, USA), which has a sensitivity of 50 IU/ml. Samples with detectable HCV-RNA were again genotyped by in-house RT-nested PCR and RFLP analysis and the RT-PCR assay (Amplicor HCV Monitor, Roche, NJ, USA) with data expressed as IU/ml.

### 2.3. Measurement of Serum Transaminases (ALT/AST)

The activity of ALT and AST was determined in serum samples collected by venipuncture using the ALT and AST test (Abbott Laboratories, Chicago, IL), and the data were reported as international unit (IU)/l units.

### 2.4. Staging of Liver Disease

The analysis of liver disease status was determined by the fibrosis-4 index (FIB-4), a noninvasive method that evaluates the state of fibrosis or cirrhosis.

### 2.5. Biological Samples

Blood samples were collected by venipuncture in tubes with a vacuum collection system. Two 5 ml tubes were collected from each patient: one tube containing EDTA anticoagulant (BD Vacutainer® EDTA K2, Franklin Lakes, New Jersey, USA) for performing the phenotypic analysis of circulating leukocytes by flow cytometry and a tube containing gel-free anticoagulant (BD SST® Gel Advance®, Franklin Lakes, New Jersey, USA) to obtain serum used for quantifying the levels of circulating cytokines and chemokines by Cytometric Bead Array (CBA).

### 2.6. Immunophenotypic Analysis of Whole Blood Leukocytes

A 100 *μ*l aliquot of EDTA whole blood was incubated in the presence of fluorescently labeled anti-human CD3-PercP, anti-CD4-PE, anti-CD8-FITC, anti-CD69-APC, anti-CD5-FITC, anti-CD19-PE, anti-CD3-PercP, anti-CD16-FITC, anti-CD56-PE, anti-CD14-APC, anti-CD80-anti-CD11c-PE, anti-CD123-FITC, anti-CD4-FITC, and anti-CD25-PercP to identify subsets of T helper, cytotoxic, T regs, NKT cells, monocytes, myeloid dendritic cells, and plasmocytes. After incubation, the cells were treated with 1 ml of erythrocyte lysis solution for 10 minutes at room temperature. After a washing step with PBS, the cells were fixed in fixed MIF solution (10 g/l paraformaldehyde, 10.2 g/l sodium cacodylate, and 6.63 g/l sodium chloride, pH 7.2). The stained cells were stored at 4°C until 24 h prior to acquisition of flow cytometry. A total of 10,000/100,000 events were purchased for each blood sample to quantify cell subsets. The FACSCalibur dual-laser flow cytometer (488 nm and 633 nm) was used to acquire and store data as FCS files. FlowJo software (Tree Star Inc., Ashland, OR, USA) was used for data analysis. Results were expressed as a percentage of positive cells.

### 2.7. Analysis of Chemokines and Cytokines

The chemokines (MCP-1 (CCL-2), IL-8), RANTES (CCL-5), IP-10 (CXCL-10) and MIG (CXCL-9), and cytokines (IL-2, IL-6, IL-10, IL-17A, IFN-*γ*, and TNF) were analyzed by the Cytometric Bead Array CBA technique, with the BD™ Human Chemokine and the BD™ Human Th1/Th2/Th17 Cytokine Kits, according to the manufacturer's specifications. For the acquisition and storage of data, the same cytometer and matrix software were used. Data were expressed as mean fluorescence intensity (MIF) for each serum chemokine and cytokine.

### 2.8. Statistical Analyses

Comparative analysis between groups was performed to evaluate the frequency of peripheral blood cell subsets as well as serum chemokines and cytokines between groups using GraphPad Prism software version 5.0 (San Diego, CA, USA). Statistical analyses were performed by the Mann–Whitney test. For the comparisons between groups of three or more variables, the ANOVA variance was used, followed by the Kruskal-Wallis test and Dunn's Multiple Comparison posttest. *P* < 0.05 was considered statistically significant in all cases.

## 3. Results

### 3.1. Study Population

This study included 35 samples from patients before treatment (pretreatment) and 35 samples from patients 12 weeks after the end of treatment (posttreatment). In addition, 50 samples from uninfected individuals framed as a healthy donor (HD) group were used as reference values for subpopulations of cells and serum concentrations of chemokines and cytokines.  Table 1 shows the demographic data and initial clinical characteristics of patients with pretreatment chronic hepatitis C (BT) and healthy donors (HD).

The group of patients with hepatitis C had a mean age group that was significantly higher than that of the HD group (60.43 ± 11.69 and 32.42 ± 11.75, respectively), with a value of *P* < 0.0001. Regarding gender, we observed that both patients with HCV infection and HD group had a predominance of male gender (*n* = 18/36), respectively.

Most patients were infected with HCV genotype 1, followed by genotypes 3 and 2. In addition, the presence of genotype 4 [8] in our region as well as the presence of genotypes 1 and 2 in a single patient was observed. It is noted that most HCV patients had a <F2 grade of hepatic fibrosis according to the classification of the FIB-4 score. Furthermore, the patients did not show significant risks of more advanced liver disease as classified by the Child-Pugh score, in which the higher frequency was classified as class A.

It is worth noting that patients with advanced hepatic injury and experience of treatment had priority in receiving the therapy containing direct-acting antiviral agents. However, most hepatitis C patients enrolled in the study were naive patients and completed 12 or 24 weeks of therapy, and none of the patients discontinued treatment.

Among the available therapeutic options, it was observed that most patients were treated with Sofosbuvir+Daclatasvir (SOF+DCV), Sofosbuvir+Simeprevir (SOF+SMV), and Sofosbuvir+Ribavirin (SOF+RBV). A 59-year-old female presented a detectable viral load at the end of 12 weeks of therapy with Sofosbuvir+Simeprevir. However, this patient underwent a second course of treatment with Sofosbuvir+Daclatasvir for 24 weeks, to which she successfully achieved SVR. One 60-year-old patient presented detectable RNA-HCV at the end of antiviral therapy, which was characterized as recurrence.

### 3.2. Hematological and Biochemical Parameters Improve after DAA Therapy

In our study, the SVR rate was reached in 94.3% of the patients. However, nonresponse to treatment was observed in two patients infected with genotype 1 and fibrosis ≥ F2. In addition, ALT/AST liver parameters were elevated before and after antiviral therapy when compared to baseline values. Despite this, there was improvement in the serum levels of these transaminases after antiviral therapy with DAAs.

Table 2 shows the hematological and biochemical characteristics of patients pre treatment (BT) and post treatment (AT) and healthy donors (HD). The hematological characteristics showed a significant decrease in the parameters of patients infected with HCV before and after treatment compared to the HD, such as the number of total leukocytes (WBC), red blood cells (RBC), hemoglobin (Hb), hematocrit (Ht), mean hemoglobin concentration (CHCM), and platelet count (PLT). Laboratory parameters associated with hepatic function were assessed at the baseline and after treatment. It is noteworthy that improvements were observed in the AT, alanine aminotransferase (ALT), aspartate aminotransferase (AST), and bilirubin patients.

### 3.3. Follow-Up of the Production of Immunological Soluble Molecules and Cell Profile in Chronic Hepatitis C Naive and Experienced Patients

**Figure 1:**
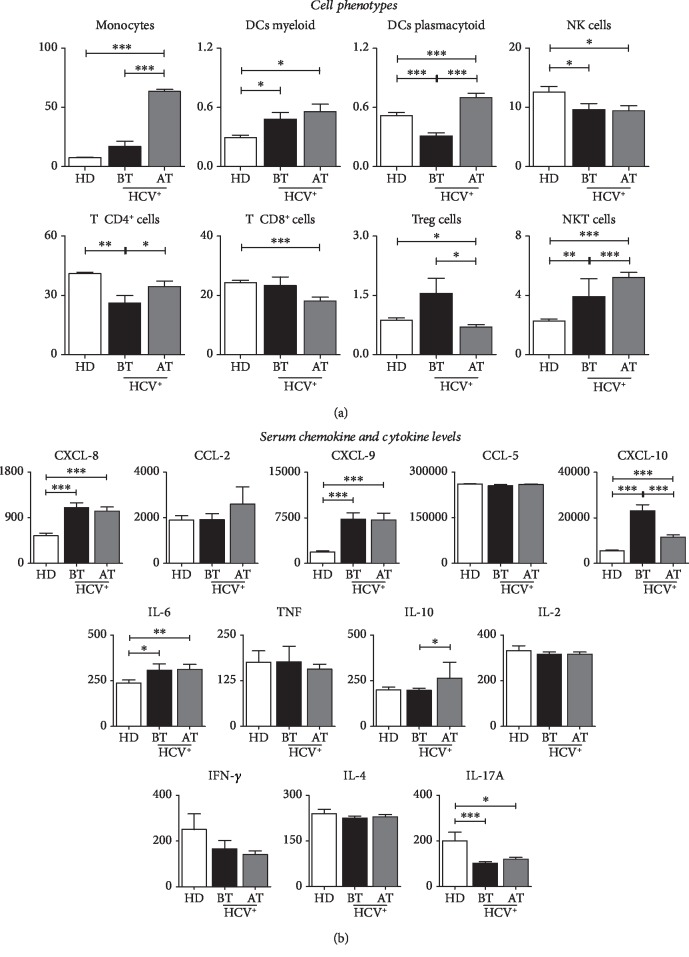
(a) Biomarkers of peripheral blood in patients with HCV and HD group. Frequency of circulating cell subunits (monocytes, myeloid dendritic cells, plasmacytoid dendritic cells, NK cells, NKT cells, CD4^+^ T cells, TCD8^+^, and T reg) in patients with HCV (before and after treatment) and HD group. (b) Serum levels of chemokines (CXCL-8, CXCL-CCL-5, and CCL-2) and cytokines (IL-2, IL-4, IL-6, IL-10, IL-17A, TNF, IFN-*γ*, and TNF) in patients with HCV (before and after treatment) and HD group. Data are expressed as the mean ± standard deviation for the percentage of cells for the subsets of circulating cells or serum concentration for chemokines and cytokines (MFI). Statistical analyses were performed by the Mann–Whitney test for comparisons between groups. Significant differences in *P* < 0.05 between HCV (before or after treatment) and CN are represented by connection lines. Differences between HCV subgroups (before or after treatment) compared to CN are highlighted by “∗”.

In order to characterize an immunological profile in patients with chronic hepatitis C before and after treatment, we evaluated the serum levels of cells, chemokines and cytokines that are presented in Figure 1. The analysis of the immune mediators demonstrated that patients with hepatitis C presented immune restoration after antiviral therapy when compared to pretreatment and control patients (Figure 1).

HCV patients after treatment exhibit high frequency of monocytes, myeloid dendritic cells, plasmacytoid dendritic cells, CD4^+^ T cells, and NKT cells (Figure 1(a)), together with a high concentration of CXCL-8, CXCL-9, and CXCL-10 chemokines and IL-6, IL-10, and IL17A modulated/proinflammatory cytokine pattern when compared to the HD group (Figure 1(b)), while NK cells, CD8^+^ T cells, and T reg cell serum concentrations decreased after the end of treatment when compared to the HD group (Figure 1(a)).

**Figure 2:**
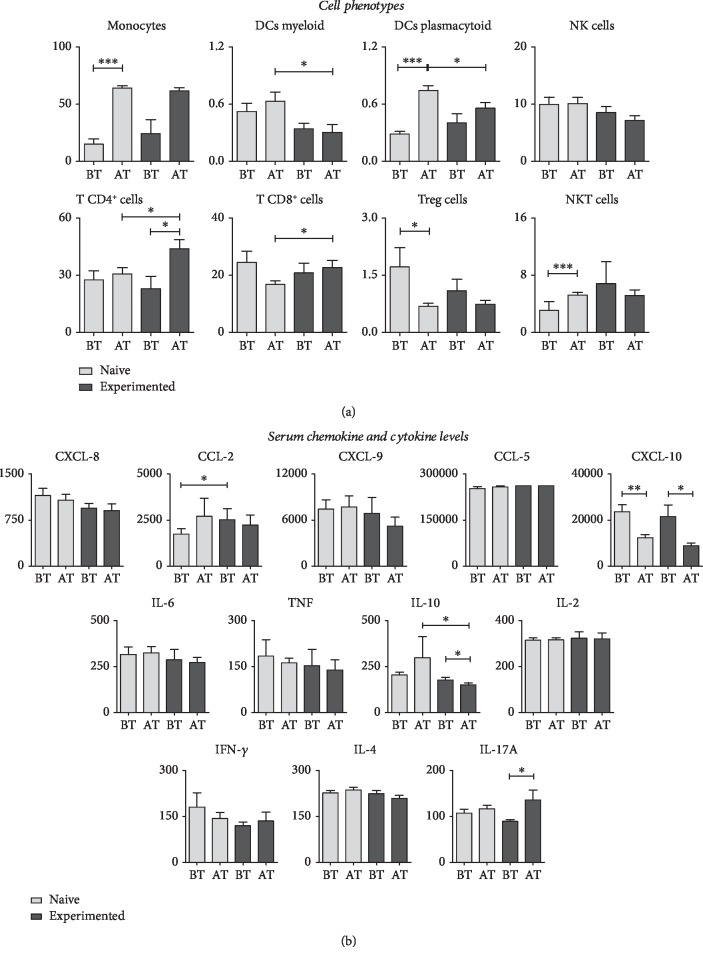
(a) Peripheral blood biomarkers in naive and experienced patients (before and after treatment). Frequency of circulating cell subsets (monocytes, myeloid dendritic cells, plasmacytoid dendritic cells, NK cells, NKT cells, CD4^+^ T cells, TCD8^+^, and T reg) in patients with HCV (before and after treatment). (b) Serum chemokines (CXCL-8, CXCL-CCL-5, and CCL-2) and cytokines (IL-2, IL-4, IL-6, IL-10, IL-17A, TNF, IFN-*γ*, and TNF) in patients with HCV (before and after treatment). Data are expressed as the mean ± standard deviation for the percentage of cells for the subsets of circulating cells or serum concentration for chemokines and cytokines (MFI). Statistical analyses were performed by the Mann–Whitney test for comparisons between groups. Significant differences in *P* < 0.05 between HCV (before or after treatment) and naive and experienced patients are represented by connecting lines. Differences between HCV subgroups (before or after treatment) compared to naive and experienced patients are highlighted by “∗”.

To determine whether immunological biomarkers of peripheral blood in patients with hepatitis C differ in naive and experienced patients before and after treatment, we evaluated the frequency of the major cell phenotypes shown in Figure 2(a). The analysis showed that after the antiviral treatment, the naive and experienced patients presented high frequency of monocytes, plasmacytoid dendritic cells, and CD4^+^ T cells. For CD8^+^ T cells, only the experienced group of patients had increases in this type of cell. As for T reg cells, both groups decreased after treatment (Figure 2(a)). Serum levels of chemokines and cytokines were found to decrease after treatment for both the naive group and the experienced group. Statistical differences were not observed in serum concentrations of cytokines, except IL-10 and IL-17A, which increased after treatment in naive and experienced patients, respectively (Figure 2(b)).

### 3.4. HCV Patients Showed Complex Biomarker Networks with Rich Interactions between Cell Profile, Chemokines, and Cytokine Production before and after Treatment

**Figure 3:**
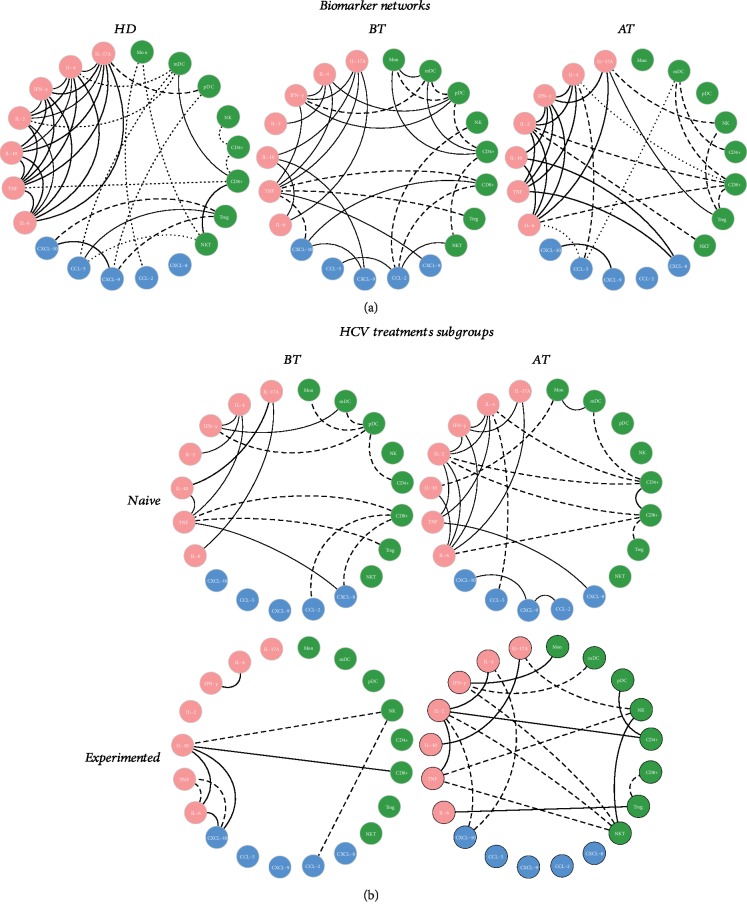
(a) Biomarker networks in naive and experienced HCV patients (before and after treatment) and the HD group. (a) Biomarker networks of the HD group and HCV patients before and after treatment. Frequency of circulating cell subsets (monocytes, myeloid dendritic cells, plasmacytoid dendritic cells, NK cells, NKT cells, CD4^+^ T cells, TCD8^+^, and T reg), serum chemokines (CXCL-8, CXCL-CCL-5, and CCL-2), and cytokines (IL-2, IL-4, IL-6, IL-10, IL-17A, TNF, IFN-*γ*, and TNF). (b) Biomarker networks of naive and experienced HCV treatment subgroups (before and after treatment). Frequency of circulating cell subsets (monocytes, myeloid dendritic cells, plasmacytoid dendritic cells, NK cells, NKT cells, CD4^+^ T cells, TCD8^+^, and T reg), serum chemokines (CXCL-8, CXCL-CCL-5, and CCL-2), and cytokines (IL-2, IL-4, IL-6, IL-10, IL-17A, TNF, IFN-*γ*, and TNF). Data are expressed as the mean ± standard deviation for the percentage of cells for the subsets of circulating cells or serum concentration for chemokines and cytokines (MFI). Statistical analyses were performed using the Mann–Whitney test for comparisons between groups. Significant differences in *P* < 0.05 between HCV (before or after treatment) and CN are represented by connection lines. Differences between HCV subgroups (before or after treatment) compared to CN are highlighted by “∗”.

Data analysis demonstrated that BT and AT have an immunological soluble molecule network different to that exhibited by the HD group (Figure 3(a)). Before treatment, correlations in the naive group demonstrated a mix of inflammatory response occurring with moderate correlations between chemokines, inflammatory cytokines, and Th2 profile, with strong regulation between IL-10 and IL-17A. Negative correlations between cell profiles and inflammatory profile are noted in this group. On the other hand, experienced patients demonstrated a poor interaction between cytokines, chemokines, and cells with a strong correlation between IL-10, IL-6, CXCL-10, and CD8^+^ besides the interactions between IFN-*γ* and IL-4. After treatment, most correlations are maintained in the naive group with a strong correlation between CD4^+^ and CD8^+^ and moderate correlation between CXCL-9, CXCL-10, and CCL-2. The experienced group began a more evident regulatory response, with increasing interaction of chemokines and regulatory cytokines, besides the presence of markers in the Th1, Th2, and Th17 profiles. In addition, a different profile was observed with a wide range of negative correlations with cytokine profiles and antiviral cell profile that characterizes an infection resolution (Figure 3(b)).

## 4. Discussion

Direct-acting antiviral treatment for chronic HCV infection may lead to complete eradication of the virus. However, little is known about the immunological changes in patients with chronic hepatitis C during and after treatment with DAAs. In general, our study explored the clinical and immunological changes in patients diagnosed with hepatitis C virus treated with distinct peripheral cell frequency profiles prior to treatment and 12 or 24 weeks after the end of treatment.

In our study, we found the presence of HCV genotypes 1, 2, 3, and 4. Genotype 4 is uncommon in Brazil, being found mainly in African countries [7]. The presence of two HCV genotypes in the same patient (1/2) was also found in this study and described by Araújo et al. [9]. This is noteworthy since it suggests that the presence of the two genotypes did not interfere in the response to therapy based on IFN-RBV. Similarly, treatment with DAAs was effective for the genotype 1 and 2 patient found in our study, since he maintained SVR after the end of treatment.

In general, patients in this study who started treatment showed reductions in Hb, Ht, MCV, and PLT levels, which did not interfere with SVR. Regarding the evolution of the treated group, the difference is remarkable in those indices that tend to stabilize after the treatment and present values close to the baseline, corroborating the data found by Zeuzem et al. [10]. On the other hand, levels of liver enzymes AST and ALT showed lower levels after the end of treatment. Corroborating our data in a retrospective analysis indicates that patients who obtained SVR tended to have lower ALT levels than patients with no response, which seems to be a natural consequence [11]. According to Saraiva et al. [12], the overall downregulation of fibrotic and inflammatory biomarkers is accompanied by an improvement in liver function, which could be observed by an expressive decrease in a laboratory parameter, such as ALT and AST, and scores noninvasive APRI and FIB-4.

During chronic HCV infection, monocytes/macrophages play an important role in triggering the adaptive immune response and in the influence of Th1/Th2 polarization, producing inflammatory and immunomodulatory cytokines. These cytokines may also impair the ability of antigen-presenting cells to activate virgin T cells and thereby assist in HCV replication and establish persistent infection [13].

In addition to monocytes, Natural Killer (NK) cells are key components of the antiviral immune response and act between innate, adaptive, and antitumor immunities. In addition, they mediate the antiviral effects producing antiviral (IFN-*γ*) and immunomodulatory (IL-10) cytokines [14, 15]. Natural Killer type T (NKT) cells are a sublineage of T cells which regulate the activation of different cell types of the innate immune system, such as macrophages, NK cells, and dendritic cells, as well as effector T cells of the adaptive immune system, with the ability to rapidly secrete large amounts of MIP-1*β* chemokines and Th1 and Th2 cytokines, including IFN-*γ* and IL-4, respectively [16].

Recently published studies suggest that NK cells may contribute to HCV clearance during therapy with DAAs. However, what is expected is that the DAAs do not have a direct effect on the phenotype, and function of these cells may result in decreased endogenous IFN-*α* and decreased activation of the NK cells [16, 17]. Interestingly, after virus elimination, Spaan et al. demonstrated reduced levels of chemokines (CXCL-10 and CXCL-11) and inflammatory cytokines (IFN-*γ* and TNF-*α*) and the late effect on cell immune systems [18]. Ahlenstiel et al. observed a decrease in the NK cell population, suggesting that this reduction may have been caused by the activation of NK cells residing in the liver or by recruitment of these peripheral blood cells to the site of infection through chemokine signaling [19].

Dendritic cells (DCs) play an important role during HCV infection; one of the most vital strategies of the HCV virus is to inhibit or interfere with the function of these antigen-presenting cells, resulting in the reduction of virus-specific T cell activation. These effects may explain or contribute to the low eliminatory capacity of the HCV immune system observed in chronically infected patients, since it is seen that these factors can probably lead to chronicity of the disease [20]. Regarding our results, we observed an increase in the frequency of myeloid DCs in patients with chronic hepatitis C after treatment. Data from literature suggest that the function of these cells is defective in patients with chronic HCV [21]. In this respect, the dysfunctionality and reduction of circulating dendritic cells in the peripheral blood of chronically infected individuals may compromise the ability of these patients to induce an effective antiviral immune response [22]. In addition, this substantial reduction was also seen during acute hepatitis C virus infection, demonstrating that the low frequency of mDC at the onset of infection may be an important factor involved in the chronicity of the disease, since these cells are efficient in migrating to the lymph nodes, present antigens to the T lymphocytes, and induce the activation and proliferation of these cells [23]. These data corroborate those found by Rana et al. [24, 25] in response to therapy with PEGIFN and RBV.

Indeed, plasmacytoid DCs (pDCs) are a specialized subset of DCs that rapidly produce a large amount of type I IFN in response to viral infections. In our study, patients with chronic hepatitis C had reduced ratios of pDCs before starting treatment. However, we observed that after successful antiviral therapy, pDCs showed high frequencies after treatment.

In the analysis of peripheral blood, CD4^+^ T cell phenotypes observed a low frequency of these cells in patients with advanced liver fibrosis (F3-4) [26]. Low CD4^+^ T cell counts may contribute to viral persistence. In addition, we can also observe in our data the CD4^+^ T cells and indicate some recovery/reversibility after treatment with DAAs. We may speculate that the decrease in the viral load induced by the treatment may allow an effective reestablishment of HCV-specific immune cell response. This contrasts with what is seen in PegIFN-based therapy, which demonstrates that T cell function is not restored during and after chronic HCV infection [27, 28]. Many studies still need to be done to elucidate this hypothesis, as it is tempting to speculate that T cell recovery has been achieved with IFN-free regimes and may have contributed to high sustained cure rates [29].

Cytotoxic T lymphocytes (CTL) act in conjunction with CD4^+^ T lymphocytes and are widely effective in antiviral control [30]. Studies have shown a robust and persistent CD8^+^ T cell proliferation in individuals with resolved HCV infection. In addition, people with acute hepatitis who have strong, well-directed CTL responses successfully control HCV infection [30, 31]. However, CTLs are believed to contribute to inflammation and liver damage during disease. [31, 32]. Nevertheless, CTLs are believed to contribute to inflammation and liver damage during the disease. In chronic HCV infection, HCV-specific CTLs present a depleted and proapoptotic phenotype according to the level of viremia, characterized by impaired proliferation, low cytotoxicity, and interferon-*γ* secretion. The decrease in induced viral load may restore a reactive HCV-specific CTL response during therapy, culminating in a high sustained response rate [33]. Our results showed that there was a strong increase of activated CD8^+^ T lymphocytes in the peripheral blood of the patients at the beginning of the treatment with subsequent decrease of these cells after the treatment. We speculate that the increase of CTL activated at the start of therapy resulted in decreased viremia and SVR induction. Data from the literature demonstrate that, in persistent HCV infection, there is a substantial reduction of specific CD8^+^ cells after virus elimination and that, in chronically infected individuals, it is not common to find a strong response of CD4^+^ cells in a sustained manner, and this differs in those individuals who eliminate the virus spontaneously [34].

It can be noted that the treatment based on DAAs presents CTL restoration specifically for HCV and has an excellent positive predictive value in the development of a sustained response. Therefore, it is speculated that after the resolution of HCV, CTL plays an essential role in the maintenance of SVR and complete eradication of HCV, preventing the risk of viral relapse [34]. In this context, Martin et al. reported that during treatment with DAAs, CTLs show restoration of the HCV-specific response and correlate with sustained viral response after successful treatment. Generally, during chronic HCV infection, there is a balance between infection control and hepatitis C immunopathogenesis, which prevents serious complications [27]. There have been studies confirming the hypothesis that T reg cells may play a protective role in controlling chronic inflammatory response and liver damage in chronic HCV carriers [34].

In chronic HCV infection, T reg cells act to suppress the inflammatory response by direct contact with effector T cells specifically for HCV and also in the production of anti-inflammatory regulatory cytokines such as IL-10 and TGF-*β* [35]. There is evidence that a high percentage of circulating T reg cells in patients with chronic hepatitis C secretes IL-10 and TGF-*β*, when compared to the group that eliminated the virus spontaneously and the HD group. This fact may explain T reg-mediated suppressor activity, promoting a poor T cell response specific to HCV and persistence of infection [36].

The results found in our study showed that the altered environment of inflammatory and anti-inflammatory cytokines of naive and experienced patients did not normalize after viral elimination, although the levels of most of the pro- and anti-inflammatory parameters were lower in naive patients when compared to the experienced patients, who in turn remained at the same level after treatment. It is important to note that the profile presented did not affect SVR. These findings corroborate those of Hengst et al. who demonstrated that HCV appears to control the microenvironment of soluble inflammatory mediators, even after viral elimination. In addition, it has been seen that even after virus clearance, complete immune restitution does not occur [37].

The increase in the expression of hepatic and peripheral CXCL-8 correlates positively with the increase of TNF-*α* and with the advancement of fibrosis, since the pronounced increase of CXCL-8 is observed in patients with a higher degree of neutrophil infiltration, cirrhosis, and impaired hepatic function [34]. In our study, there was a significant increase in CXCL-8 and IFN-*γ* concentrations in HCV-infected patients when compared to the healthy donors. Corroborating with our results, similar data were described by Han et al. in patients treated with PEGIFN and RBV [38]. CCL-2 is an important chemokine involved in the recruitment of monocytes/macrophages and T cells to areas of inflammation, with high prooncogenic action regulated in the liver, playing crucial roles in recruitment of monocytes and activation of HSC in the induction of hepatic fibrosis. Elevated circulating levels in serum and inflamed tissue have been associated with rapid progression to liver failure in patients with HCV [39].

HCV interferes with cytokines at various levels and avoids immune response by inducing a Th2 or fibrogenic cytokine profile. In a study of populations of patients with chronic hepatitis C with different degrees of fibrosis, Souza-Cruz et al. [26] found that the concentration of IL-10 and IL-6 was elevated only in individuals in the <F2 moderate fibrosis group in relation to the ≥F2 advanced fibrosis group, suggesting protective and anti-inflammatory activity in moderate fibrosis group <F2. On the other hand, it has been seen that patients with chronic hepatitis C treated with PEGIFN+RBV and presenting an increase in IL-10 regulatory cytokine may actually compromise the host immune response [38].

In our cohort of naive patients, there was a statistically significant change in IL-10 after treatment with DAAs, whereas experienced patients showed elevated levels of IL-17A after treatment with DAAs. The increase in Th17 cells and elevated levels of IL-17 are also associated with severe hepatic damage, since these cytokines participate in tissue repair and recruitment of inflammatory cells during Th1 responses. It has been seen that lower immune responses of the Th17 profile are accompanied by a less fibrogenic environment. Therefore, we believe that it is fundamental not only to evaluate these patients as SVR but also to evaluate the restoration of the immunological profile in order to detect changes which are suggestive of progressive involvement in the hepatic tissue through serum biomarkers.

The study had some limitations regarding the size of the sample due to the short period of recruitment and due to the spontaneous demand of patients.

## 5. Conclusion

In conclusion, our data provide evidence that viral clearance induced by interferon-free therapy leads to improvement in liver function. However, it does not lead to complete reversibility of cellular profile accompanied by the chemokine and cytokine profile at 12 and/or 24 weeks after the end of treatment and may be related to the presence of established liver disease. In addition, naive and experienced patients seem to have a distinct soluble biomarker profile; therefore, a long-term follow-up is needed to address the important question regarding the parameters in patients successfully treated with DAAs.

## Figures and Tables

**Table 1:** Demographic and clinical characteristics of the control group and HCV patients before treatment.

	Control group (*N* = 50)	HCV patients (*N* = 35)	*P* value
Demographics & clinical characteristics			
Age (mean ± SD^∗^)	32 ± 12	60 ± 12	**<0.0001** ^**#**^
Gender (male/female)	36/14	18/17	*—*
HCV genotypes (1/2/3/4/1+2)^∗^	—	25/2/6/1/1^∗^	—
Naïve/experienced	—	27/8	—
≤F2 (FIB‐4 < 3, 25)	—	18/17	—
>F2 (FIB‐4 > 3, 25)	—	17/18	—
Child-Pugh A/B/C	—	31/2/2	—
Hypertension portal (yes/no)	—	8/27	—
DAA treatment			
SOF+SMV	—	5	—
SOF+DCV	—	23	—
SOF+RBV	—	2	—
12/24 weeks	—	22/13	—
SVR/no SVR	—	34/1	—

^#^Mann–Whitney test; SD: standard deviation; ^∗^genotype: patient denied viral load prior to treatment. Patients present two HCV genotypes: naïve: patients without previous treatment, and experienced: patients with more than one therapy. FIB-4: noninvasive score to determine hepatic fibrosis; FIB‐4 > 3.25 features METAVIR ≥ F2. Portal hypertension: criteria for evaluation of portal hypertension: esophageal varices, thrombocytopenia, and splenomegaly; SOF/SMV: Sofosbuvir+Simeprevir; SOF/DCV: Sofosbuvir+Daclatasvir; SOF/RBV: Sofosbuvir+Ribavirin; SVR: sustained virological response; no SVR: no sustained virological response.

**Table 2:** Hematological and biochemical characteristics of the control group, before (BT) and after (AT) treatment.

Characteristics	Control group (*N* = 50)	HCV patients		*P* value
		BT (*N* = 35)	AT (*N* = 35)	
Hematologic (mean ± SD)				
WBC (unid.×10^3^/mm^3^)	6.2 ± 1.4	5.7 ± 3.0	5.1 ± 1.6	**<0.0043** ^**a**^ **<0.0012** ^**b**^
RBC (unid.×10^6^/mm^3^)	5.1 ± 0.6	4.4 ± 0.8	4.5 ± 0.6	**<0.0001** ^**a,b**^
Hb (g/dl)	15.1 ± 1.8	13.7 ± 2.2	13.3 ± 1.6	**<0.0003** ^**a**^ **<0.0001** ^**b**^
Ht (%)	44.5 ± 5.6	40.3 ± 6.5	39.6 ± 4.3	**<0.0001** ^**a,b**^
MCV (fl)	87.4 ± 4.4	89.6 ± 6.2	88.9 ± 6.9	0.2033^a,b^
MCH (pg)	29.6 ± 1.6	30.5 ± 2.4	29.8 ± 2.7	0.1105^a,b^
MCHC (g/dl)	34.0 ± 1.7	33.9 ± 1.3	33.6 ± 1.1	0.1661^a,b^
RDW (%)	13.5 ± 0.7	13.7 ± 1.1	13.9 ± 1.1	0.2836^a,b^
PLT (unid.×10^6^/mm^3^)	247.6 ± 55.7	165.9 ± 83.9	186.1 ± 83.6	**<0.0001** ^**a,b**^

Biochemical (mean ± SD)				
	Reference value^∗∗^	BT (*N* = 35)	AT (*N* = 35)	*P* value
AST (UI/l)	5-40	54.3 ± 37.6	36.9 ± 26.2	0.0660
ALT (UI/l)	10-55	57.4 ± 46.1	31.2 ± 25.3	**0.0162**
BT (mg/dl)	0.1-1.2	1.0 ± 0.7	0.9 ± 0.6	0.7403

^#^Kruskal-Wallis test; SD: standard deviation; WBC: white blood cells; RBC: red blood cells; Ht: hematocrit; Hb: hemoglobin; MCV: mean corpuscular volume; MCH: medium corpuscular hemoglobin; CHCM: mean corpuscular hemoglobin concentration; RDW: red cell distribution width; PLT: platelet; AST: aspartate aminotransferase; ALT: alanine aminotransferase. ^a^Comparison of the control group with BT patients; ^b^comparison of the control group with AT patients, nonparametric *t*-test application and Mann–Whitney. Reference value ∗∗: retrieved from results of biochemical tests of kits.

## Data Availability

The data used to support the findings of this study are included within the article.
